# The art of passing unnoticed: pathogenic fungi remain incognito thanks to EWCA effectors

**DOI:** 10.1093/plcell/koab017

**Published:** 2021-01-25

**Authors:** Valentin Hammoudi

**Affiliations:** Institute of Biology, Applied Genetics, Freie Universit�t Berlin, Albrecht-Thaer-Weg 6, 14195 Berlin, Germany

Some are better at avoiding than managing conflicts. Such is the case for the cucurbit powdery mildew fungus *Podosphaera xanthii*, which passes unnoticed during infection thanks to its Effectors with Chitinase Activity (EWCAs).

Plants sense the presence of potentially harmful microbes via the recognition of specific molecules called microbe-associated molecular patterns (MAMPs; also known as pathogen-associated molecular patterns). Typically, MAMPs are well-conserved microbial molecules and are recognized by plant cell surface proteins named pattern recognition receptors (PRRs). Once PRRs detect micro-organisms, the plant immune system springs into action: toxic compounds such as reactive oxygen species accumulate and plants turn on hundreds of defense-associated genes (Newman et�al., 2013). To overcome these defenses and successfully invade their hosts, pathogens and symbionts have evolved a wide range of effectors that inhibit the plant immune response at different levels. One outstanding strategy is to block detection at the MAMP recognition level, essentially flying “under the radar”.

Chitin, a component of the fungal cell wall, is the best-characterized MAMP produced by fungi. Chitin itself is not directly recognized by plant cells, but is broken down by plant-secreted chitinases into chitin oligomers. The plant PRR CHITIN ELICITOR RECEPTOR KINASE 1 (CERK1) recognizes the fragmented chitin and activates plant defenses. To avoid the elicitation of the chitin-triggered response, fungi have evolved different strategies (S�nchez-Vallet et�al., 2013). For example, some fungi secrete chitin deacetylases that transform chitin into chitosan, a compound that cannot be processed by plant chitinases. Alternatively, other fungi species secrete proteins that bind to chitin or chitin oligomers, hiding them from plant chitinases or chitin-receptors.

In this issue, Jes�s Mart�nez-Cruz et�al. (2021) shed light on the strategy employed by the cucurbit powdery mildew fungus *P. xanthii*, a biotrophic pathogen, to overcome the chitin-triggered immune response. As a starting point, the authors scanned the genome of *P. xanthii* and identified several genes encoding multiple Podosphaera Effector Candidates (PECs). These genes encode uncharacterized proteins with an identical signal peptide at the N-termini. Additionally, the induction of these *PECs* correlates with the establishment of primary haustoria (feeding structures) during fungal infection of leaves. Combining protein modeling and protein–ligand prediction, the authors revealed predicted structural similarities between PECs and chitinases. In line with this, some of these PECs displayed chitinase activity *in vitro*. Subsequently, these PECs were renamed as “Effectors with Chitinase Activity” or EWCAs.

Using an elegant host-induced gene silencing approach in melon plants inoculated with *P. xanthii*, the study then established that downregulation of *EWCAs* caused reduced fungal growth and a stronger activation of plant immunity compared with control assays. Strikingly, this immune response relies almost entirely on the perception of chitin via the plant receptor CERK1, as co-silencing of both fungal *EWCAs* and the plant *CERK1* led to a similar pattern to control plants infected by *P. xanthii*. Using for the very first time, the “transformation by growth on agro‐infiltrated tissues” tool (Mart�nez-Cruz et al., 2018) to express GFP-effector fusions in powdery mildew cells, confocal microscopy confirmed that EWCAs localize at early stages of fungal infection to plant papilla, which is the prime penetration point of biotrophic fungi into plant tissues. Most likely, EWCAs dampen plant defenses by digesting chitin oligomers to sizes too small to be detected by CERK1. This strategy represents a novel mechanism for “disguising” MAMPs to avoid recognition by host immune receptors (see Figure�1).

**Figure 1:**
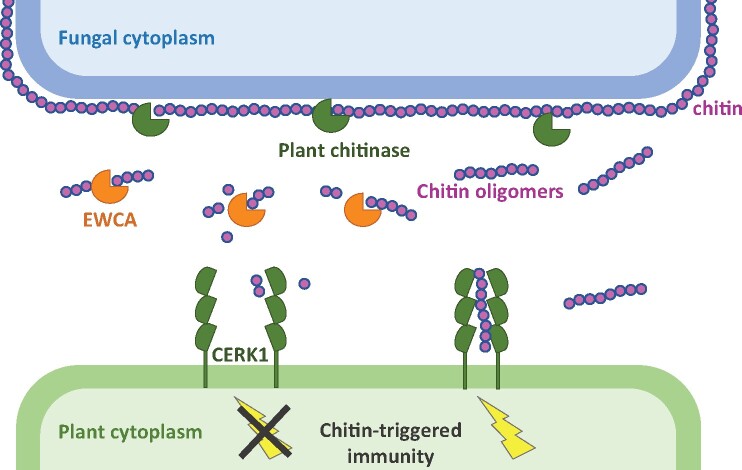
Proposed Role for EWCA effectors. At fungal pathogen penetration sites, plant-secreted endochitinases release chitin fragments from fungal cell walls that are recognized by the plant chitin elicitor receptor kinase CERK1, activating chitin-triggered immunity. To counteract this action, EWCAs are released by the fungus at the same sites to break down immunogenic chitin oligomers into smaller molecules that cannot induce dimerization of CERK1, thus suppressing the activation of chitin-triggered immunity (Adapted from Mart�nez-Cruz et�al., 2021; Figure 10).

Finally, BLAST searches revealed that EWCAs are widely distributed among fungal pathogen species, with up to nine copies present in some powdery mildew species. Remarkably, some fungal species with EWCAs are pathogenic toward insects, nematodes, or even humans. Although the specificity of each EWCA ortholog remains elusive, escaping the chitin-triggered immune response, thanks to these effectors appear to be broadly conserved among pathogens to initiate the successful infection of their host. The discovery of these novel EWCAs adds another piece to the puzzle for our understanding of how successful pathogens avoid eliciting host immune systems. 
